# Can We Simplify Aging?

**DOI:** 10.1093/geroni/igab046.2502

**Published:** 2021-12-17

**Authors:** Svetlana Ukraintseva, Anatoliy Yashin

**Affiliations:** Duke University, Durham, North Carolina, United States

## Abstract

Aging is indeed a complex process, but can it be simplified, so we could efficiently prioritize candidate anti-aging interventions and select those with largest impacts on key negative consequence of the aging, i.e., on increases in mortality risk and comorbidities with age? Here we argue that human aging and its negative consequences for health and lifespan are essentially driven by the interplay among three processes: (i) depletion of limited body reserves (e.g., of stem, immune, neural, muscle cells); (ii) inherent deficiency of cell/tissue repair mechanisms, which leads to accumulation of damage, allostatic load, and systems dysregulation; and (iii) general slowdown of physiological processes in the body (such as metabolism, proliferation and information processing) with age that results in slower responses to stressors and delayed recovery after damage (i.e., decline in resilience), which in turn contributes to increase in vulnerability to death with age. We show that the interplay among these processes can have ambivalent effects on health and longevity that should be taken into account to develop optimal anti-aging and pro-longevity strategies. In order to be efficient on the long-term, the anti-aging interventions may need to target the different causes of aging (reserve depletion, damage accumulation, and slowdown) simultaneously, to avoid undesirable trade-offs.

